# Low Genetic Diversity of Hepatitis B Virus Surface Gene amongst Australian Blood Donors

**DOI:** 10.3390/v13071275

**Published:** 2021-06-30

**Authors:** Ngoc Minh Hien Phan, Helen M. Faddy, Robert L. Flower, Wayne J. Dimech, Kirsten M. Spann, Eileen V. Roulis

**Affiliations:** 1 Faculty of Health, School of Biomedical Sciences, Queensland University of Technology, Kelvin Grove, Queensland 4059, Australia; hfaddy@usc.edu.au (H.M.F.); rflower@redcrossblood.org.au (R.L.F.); kirsten.spann@qut.edu.au (K.M.S.); eroulis@redcrossblood.org.au (E.V.R.); 2 Research and Development, Australian Red Cross Lifeblood, Kelvin Grove, Queensland 4059, Australia; 3 School of Health and Behavioural Sciences, University of Sunshine Coast, Petrie, Queensland 4502, Australia; 4 Scientific & Business Relations, National Serology Reference Laboratory, Fitzroy, Victoria 3065, Australia; wayne@nrlquality.org.au

**Keywords:** hepatitis B virus, genetic diversity, ‘a’ determinant, hepatitis B surface gene, blood donor

## Abstract

Variants in the small surface gene of hepatitis B virus (HBV), which codes for viral surface antigen (HBsAg), can affect the efficacy of HBsAg screening assays and can be associated with occult HBV infection (OBI). This study aimed to characterise the molecular diversity of the HBV small surface gene from HBV-reactive Australian blood donors. HBV isolates from 16 HBsAg-positive Australian blood donors’ plasma were sequenced and genotyped by phylogenies of viral coding genes and/or whole genomes. An analysis of the genetic diversity of eight HBV small surface genes from our 16 samples was conducted and compared with HBV sequences from NCBI of 164 international (non-Australian) blood donors. Genotypes A–D were identified in our samples. The region of HBV small surface gene that contained the sequence encoding the ‘a’ determinant had a greater genetic diversity than the remaining part of the gene. No escape mutants or OBI-related variants were observed in our samples. Variant call analysis revealed two samples with a nucleotide deletion leading to truncation of polymerase and/or large/middle surface amino acid sequences. Overall, we found that HBV small surface gene sequences from Australian donors demonstrated a lower level of genetic diversity than those from non-Australian donor population included in the study.

## 1. Introduction

Hepatitis B virus (HBV) is a leading cause of hepatic disease with an estimated 248 million chronic infections and 686,000 deaths per year globally [1,2]. The prevalence of HBV infection detected in blood donors varies by country or region, with a prevalence of 2.3% in China [3], 7.5% in Ghana [4], 2.3% in eastern Mediterranean and middle eastern countries [5] and 0.32% in Italy [6]. In 2016, Australia recorded a low rate of 0.06% HBV infections detected in total first-time blood donations [7]. 

HBV is a small, enveloped DNA virus with a relaxed circular (rcDNA) and partially double-stranded genome of 3.2 kbp. The HBV genome has four partially overlapping open reading frames (ORFs) coding for the polymerase, pre-surface/surface, pre-core/core and X proteins [8]. The pre-surface/surface coding sequences encode large, middle, and small envelope proteins which play an important role in viral attachment to hepatic cells [8]. HBV surface protein (HBsAg) is a glycoprotein of 226 amino acids (AA) and bears epitopes of human B and T cells within the major hydrophilic region (MHR) (AA99–169), which are essential for the development of human anti-HBs antibody [9,10]. HBsAg is a first-line diagnostic marker of HBV infection. The ‘a’ determinant (AA124–147) is a common neutralising epitope in HBV of all genotypes. Antibodies against this determinant can protect individuals against infection from wild-type HBV [10].

The HBV genome displays high genetic diversity. Ten HBV genotypes, A to J, are recognised with at least 8% nucleotide diversity between genotypes across the genome [11]. HBV surface and polymerase genes, and whole genomes are usually used for genotyping [12,13,14]. Compared to these genes, HBV core gene is less frequently used for genotyping given its short length. However, core gene-based phylogenies are used to investigate mixed infections, recombination, and transmission patterns of HBV [15,16].

HBV genotypes display a geographically and ethnically distinct distribution. Genotypes B and C are most frequently found in Southeast Asia, Australia and New Zealand. Genotypes A and D are most frequently found in Europe, North Africa, North America and Asia, whilst genotypes E, F, G and H are most commonly found in Sub-Saharan Africa, and Latin America [17].

Quasispecies refer to a viral population of highly related but not identical genomes [18]. HBV replication is somewhat similar to retroviruses in the reverse transcription step, which is mediated by viral polymerase [19]. Due to the lack of proofreading of the HBV polymerase, the replication process can accumulate variations, and form quasispecies during prolonged or chronic infections [18,20]. Diversity within quasispecies has been shown to be more limited in the overlapping polymerase coding region of HBV than the core gene [18]. The presence and complexity of quasispecies are associated with the development of drug resistance, viral adaptability and liver disease progression [18]. Antiviral therapy, such as the use of telbivudine, can increase the complexity level of HBV quasispecies [21]. A high prevalence of viral quasispecies was also reported in hepatocellular carcinoma [22].

A number of genetic variants in the HBV surface gene may inhibit the efficacy of commercial HBsAg diagnostic assays [23]. These variants can affect the sensitivity and specificity of screening assays for HBV if they alter HBV DNA levels or the structure of the MHR of HBsAg protein used in diagnostic tests [24,25]. One suggested reason for this is that AA substitutions within this region, especially in the ‘a’ determinant or in the MHR, such as the variants p.G145R [26] and p.Y100C [27], may alter the hydrophilicity, electrical charge and/or acidity of HBsAg protein, affecting HBV antigenicity and leading to immune escape, or false-negative detection of HBsAg by commercial tests [23].

There is little understanding of HBV types and their genetic diversity in Australian blood donors, with no publicly available surface gene sequences from Australian blood donors on the NCBI nucleotide database. Therefore, this study aimed to characterise the HBV surface gene sequences amongst Australian blood donors. Furthermore, we investigated nucleotide heterogeneity and AA variations in the HBV surface, polymerase and core genes between our samples (Australian sequences) and publicly available international (non-Australian) sequences from blood donors.

## 2. Materials and Methods

### 2.1. Plasma Samples 

A total of sixteen de-identified plasma samples were collected from Australian blood donors residing in Australia (designated HBV1-16). Fourteen samples were provided by the National Serology Reference Laboratory (NRL; Fitzroy, Victoria, Australia). Two samples were supplied from a previous study [28] by the Scientific and Technical Services of Australian Red Cross Lifeblood (Kelvin Grove, Queensland, Australia). Results of nucleic acid amplification testing (NAT) and serology testing, as well as demographic information, were provided along with the samples. Donors’ ethnicity data were not collected for this study. All donors had provided written consent for blood to be used for research purposes. This research was approved by the Human Research Ethics Committee (HREC) of Australian Red Cross Lifeblood (reference number: Faddy 12062018, approved on 12 June 2018) and the Queensland University of Technology HREC (approval number: 1800000534, approved on 20 June 2018).

### 2.2. DNA Extraction and HBV Viral Quantification

DNA was extracted from plasma (200 µL) using the QIAmp MinElute virus spin kit (QIAgen, Hilden, Germany) and eluted in AVE buffer (35 µL) as per the manufacturer’s instructions [29]. Viral loads were quantified for each sample using the HBV Genesig^®^ standard kit (Primerdesign^TM^ Ltd., Eastleigh, UK), which targets a region in the core gene [30]. Total DNA yields (ng/µL) were quantified using Qubit DNA broad range (BR) and high sensitivity (HS) assays. Further DNA extractions were performed as required and samples were normalised to at least 100 gene copies/µL for downstream applications. 

### 2.3. HBV Nested Polymerase Chain Reactions (PCRs)

For each sample, six overlapping HBV amplicons were generated using primer sets (set 1a/b, set 2a/b, set 3 and set 4) previously described by Chook et al. (2015) [31]. The primer sets were designed to amplify overlapping regions of 748–1203 bp along the full-length genome [31]. Invitrogen Platinum SuperFi PCR master mix [32] was used for the nested PCRs. Amplified products were stained with GelRed^®^ nucleic acid gel stain, 10,000× (Biotium, San Francisco, CA, USA) [33] and PCR products visualised by agarose gel electrophoresis to confirm the expected product size. Amplicons were quantified by Qubit DNA HS or BR assays. PCR products of each amplicon were pooled to a minimum of 100 ng DNA, concentrated and eluted in molecular grade water (35 µL) for library preparation [34].

### 2.4. HBV Sequencing and Preliminary Analysis

Libraries were prepared using the Illumina^TM^ DNA Prep kit (Illumina, San Diego, CA, USA) and sequenced on an Illumina MiSeq using V3 2 × 150 bp chemistry [35]. For each amplicon, the demultiplexed reads were mapped to the NCBI HBV reference genome NC_003977 and a consensus sequence generated from regions with at least 800× coverage for each sample. 

### 2.5. Genotyping by Phylogenetic Analysis

Core, polymerase and large surface genes were extracted from samples that had read depth of at least 800× at these loci. Reference genome sequences (genotypes A to H) from the NCBI HBV genotyping tool were used [36], and supplemented with genomes FJ023664 (genotype I), AB562463 (genotype I) and AB486012 (genotype J) (Table S1). The woolly monkey HBV genome AF046996.1 was used for outgrouping. Nucleotide sequences were aligned with MAFFT v7.450 [37,38] in Geneious Prime^®^ (www.geneious.com/, accessed on 4 February 2020, Biomatters Ltd., New Zealand). For genotyping, approximated maximum-likelihood trees of core, polymerase and large surface genes were built using FastTree 2.1.11 with default settings [39,40]. For samples where the whole HBV genome was obtained and phylogenetic analysis suggested possible recombinants, we investigated recombination events using the BootScanning program with default settings (100 replicates, parental threshold of 70%) built into Simplot software [41]. Consensus genotypes for all samples, including those with suspected recombination events, were then determined.

### 2.6. Comparative Analysis of Nucleotide Diversity of HBV Small Surface Gene

Nucleotide variability analysis of the HBV small surface coding region, which encodes the HBsAg, was conducted using Australian and non-Australian sequences (Table S1). Australian sequences were obtained from our samples, which were collected from blood donors living in Australia. Non-Australian sequences randomly retrievable from the NCBI database were from blood donors outside Australia. The NCBI reference sequences were genotypes A-H, with no sequences from blood donors found for genotypes I and J. From the 164 sequences retrieved from NCBI, each genotype was represented by one to maximum 30 different surface sequences, depending on their availability. Variations of nucleotide diversity (Pi values) in the HBV surface gene between Australian and non-Australian sequences were investigated using DnaSP 6, with a window length of 100 bp and step size of 25 bp [42]. The antibody epitope prediction server IEDB-AR (http://tools.iedb.org/bcell/, accessed on 5 June 2021) [43,44] was used to predict antigenic segments for translated HBsAg sequences that had no ambiguous amino acids from our samples. This was to determine the location of nucleotide regions of high genetic diversity in comparison with regions coding for two antibody epitopes of HBsAg previously predicted by Rezaee et al. (2016) [26].

### 2.7. Variant Calling

Consensus sequences of polymerase and large/middle/ small surface genes with at least 800× coverage from each sample were aligned with the NCBI HBV reference genome NC_003977. Variant calling was then conducted for these nucleotide alignments to identify synonymous and nonsynonymous single nucleotide variants (sSNV and nsSNV) inside the coding sequences. One sample with suspected quasispecies subpopulations was further investigated via analysis of called variants from viral reads mapped to reference sequence using Geneious Prime^®^ 2020.2.4. with default settings. 

### 2.8. Identification of HBV Small Surface AA Variations

Escape mutations in the HBV small surface gene from our samples were identified using the web-based HBV drug resistance interpretation tool (DRI) [45]. Additionally, translated small surface coding sequences from our samples together with additional reference of HBV A-J genotypes from NCBI (Table S1) were manually inspected for AA variations previously reported to associate with OBI [46] using Geneious Prime^®^ 2020.2.4. The OBI-related variants selected for this study included p.Y100C, p.Y100S, p.T116N, p.G119R, p.P120T, p.C121, p.R122P, p.C124, p.C124R, p.C124Y, p.I126S, p.Q129R, p.S136P, p.C137, p.C139R, p.T140I, p.K141E, p.D144A, p.G145R, p.G145A, p.Y100S + p.S143L, p.M103I + p.G145A, p.M103I + p.R122K, p.T116N + p.S143L, p.R122P + p.Q101R, p.R122P + p.S167L, and p.R122K + p.G145A [46].

## 3. Results

### 3.1. Demographic and Clinical Characteristics of Study Samples

Sample collection dates ranged from December 1997 through to May 2011. The results of HBV detection tests and collection dates were provided by Australian Red Cross Lifeblood and National Serology Reference Laboratory (Table 1). All samples were from Australian blood donors who resided in Australia and were positive to HIV-1/HCV/HBV multiplex NAT, HBV discriminatory NAT and HBsAg serology testing. A single sample was both HBV- and HCV-reactive. Samples HBV 1, 9, 10 and 11 were positive to total anti-HBc and anti-HBe, but negative to anti-HBc IgM, HBeAg and anti-HBs (Table 1).

### 3.2. HBV Viral Load and PCR Amplicon Quantification 

Total DNA and viral load for each sample were quantified using fluorometry and qPCR (Table S2). Samples HBV 3, 7, 12 and 13 had over 2000 copies/µL while samples HBV 10, 12, 15, and 16 had over 100 copies/µL. Eight samples had less than 100 copies/µL.

### 3.3. HBV Sequencing and Preliminary Analysis

**Figure 1:**
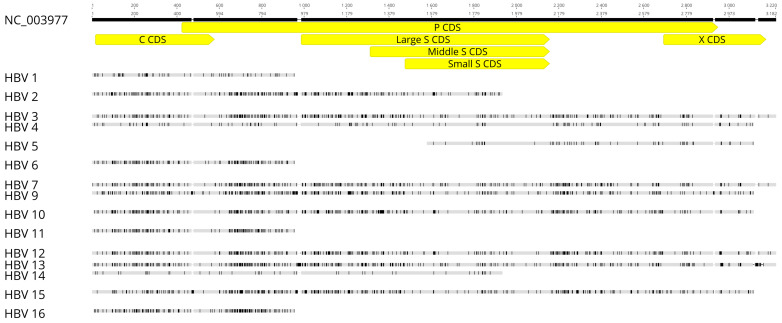
HBV sequences from our samples aligned by MAAFT program. Polymerase, core, X and surface coding sequences are highlighted in yellow boxes. Black bars highlight disagreements between the sample sequence and the reference sequence (NC_003977).

We obtained 15 partial and complete HBV sequences from 16 samples (Figure 1). Whole genomes were recovered from samples 3, 7, 12 and 13; all of these samples had viral loads greater than 2000 copies/µL (Table S2). Consensus sequences obtained from mapped reads for samples HBV 4, 9, 10 and 15 covered the majority of the HBV genome, except for a region encoding a small part of X protein and pre-core. HBV fragments generated from samples HBV 1, 2, 6, 11, 14 and 16 covered the core region and a small part of the polymerase region. Sample HBV 5 had a small HBV fragment covering partial regions of small surface, polymerase or X proteins (Figure 1). Accession numbers for these HBV sequences are MW847929–MW847943. 

### 3.4. Genotyping by Phylogenetic Analysis

Amongst the 15 HBV sequences obtained from our samples, only 14 were whole genomes or had complete polymerase, core and/or large surface genes. For each sample, the sequences extracted from these genes were genotyped by analyses of maximum-likelihood phylogenies built by FastTree (Figures S1–S4). Genotypes are described in Table 2. Genotype C was the most prevalent (6/14 sequences), followed by genotypes B, D and A.

**Figure 2:**
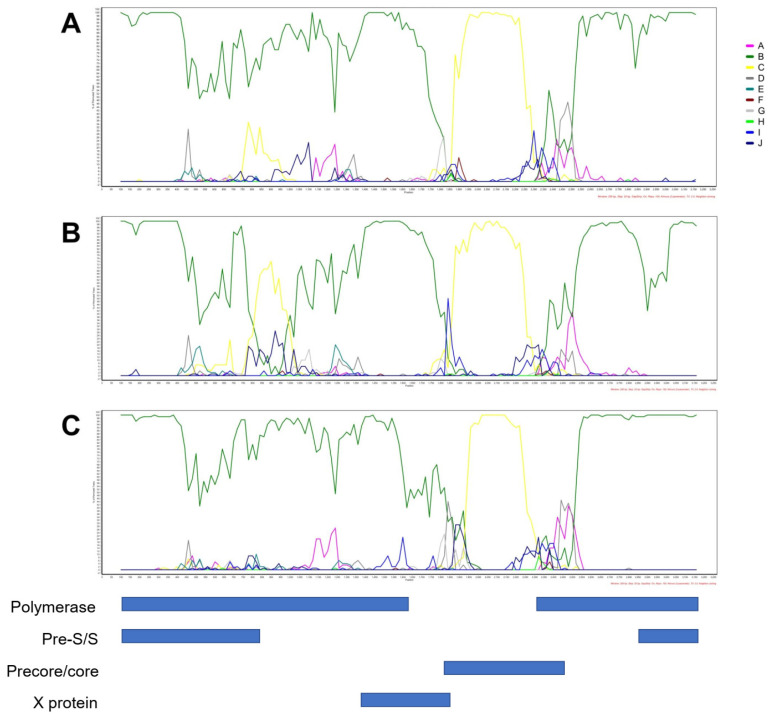
BootScan analysis over nucleotide position along the HBV genome for samples (**A**) HBV 3, (**B**) HBV 7, and (**C**) HBV 13. The y axis shows the percentage of permuted trees in which the selected HBV genotypes clustered with the query sequence. Genotypes were colour-coded.

Samples HBV 3, 7 and 13 had discrepancies in genotyping by phylogenies of HBV core and polymerase/large surface genes, suggesting potential recombination. An analysis of recombination events for samples HBV 3, 7 and 13 was therefore conducted using Simplot (Figure 2). All three samples showed similar recombination events between genotypes B and C within the HBV core gene. 

### 3.5. Analysis of HBsAg Nucleotide and AA Diversity

**Figure 3:**
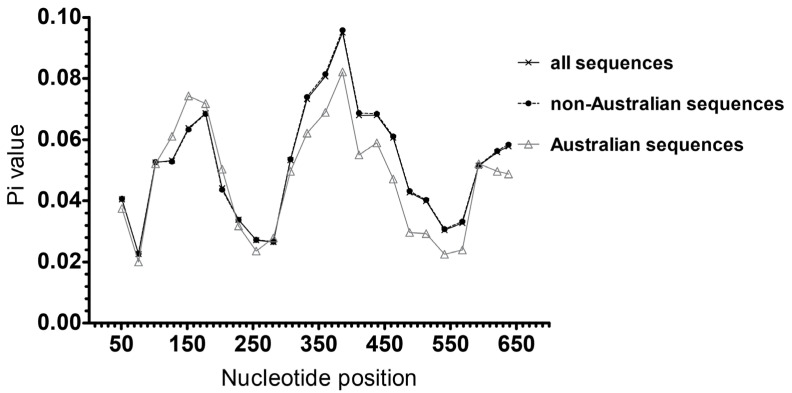
Nucleotide diversity (Pi) estimated over mid-point nucleotide position in the HBV surface gene from blood donors (window length of 100 bp and step size of 25 bp). The MAFFT-aligned sequences of surface gene were 681 bp in length.

Only eight sequences amongst the 15 HBV sequences obtained from our samples contain the small surface region. They were included in the analysis of HBsAg diversity to represent Australian sequences. A comparative analysis of nucleotide diversity of the HBV small surface gene was conducted between the eight sequences from Australian blood donors and 164 small surface gene sequences from international (non-Australian) blood donors retrieved from NCBI. There were no HBV surface gene sequences from Australian blood donors in the NCBI nucleotide database. We identified two peaks of genetic diversity in regions nt103–203 and nt333–438 (Figure 3). Diversity in region nt103–203 was higher amongst Australian sequences compared to those outside Australia, whilst diversity in region nt333–438 was slightly lower. Non-Australian HBV sequences had the trendline of Pi values identical to the line of all investigated sequences and they are hence superimposed in Figure 3. 

**Figure 4:**
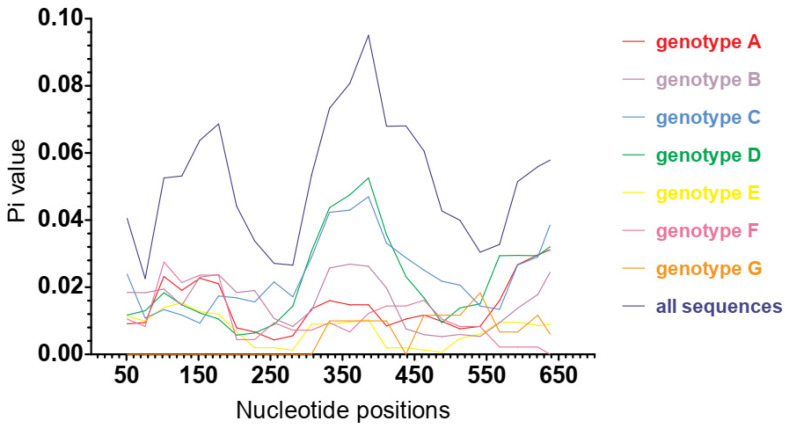
Genotypic variation of nucleotide diversity (Pi) estimated over mid-point nucleotide position in the HBV surface gene from blood donors (window length of 100 bp and step size of 25 bp). The MAFFT-aligned sequences of surface gene were 681 bp in length.

When the two surface gene regions of variability identified in Australian and non-Australian blood donors are compared by genotype, we observed that variability within the nt300–400 region was greater than the variability within the nt103–203 region (Figure 4). Interestingly the former region incorporates the sequence coding for the ‘a’ determinant (AA124–147) of HBsAg. Among eight translated HBsAg sequences from our samples, only three sequences (HBV 3, 9 and 10) had no ambiguous amino acids and were eligible for prediction of antibody epitopes with use of the server IEDB-AR [43,44]. Major antigenic segments of HBsAg from these three samples were predicted to locate within nt130–207 and within nt328–441 (Files S2–S4). 

No escape mutants and none of the selected OBI-related variants were identified in our samples (File S1). Fifteen international (non-Australian) sequences contained at least one OBI related variant: five with p.Y100C, one with p.Y100S, one with p.T116N, four with p.P120T, two with p.T140I, one with p.G145R or one with combined p.M103I and p.R122K (File S1).

### 3.6. Variant Calling

sSNV and nsSNV were identified inside the polymerase and large/middle/small surface coding sequences between the consensus sequence of each sample and NCBI reference sequence (Table 3). HBV isolates of genotypes A and B from our samples had higher total numbers of sSNV and nsSNV for polymerase and surface genes than those of genotypes C and D. 

For HBV 10 (Genbank accession number: MW847935), manual investigation indicated a frameshift mutation due to a nucleotide deletion in the overlap of the polymerase and surface genes, resulting in truncated large surface, middle surface and polymerase amino acid sequences. The deletion is located at nt905 on the polymerase gene (Figure S5), which corresponds to nt364 on the large surface gene (Figure S6), or nt40 on the middle surface gene (Figure S7).

Similarly, HBV 12 (Genbank accession number: MW847942) had a nucleotide deletion that truncated the polymerase amino acid sequence. The position nt549 of the missing nucleotide determined by mapping the reads of HBV 12 to the reference sequence was different from the position nt538 determined by consensus sequence alignment of HBV 12 gene (Figure S8), probably due to a high repetitive nature of the region with the presence of several degeneracies between the two sites. However, the nucleotide deletion at either position resulted in the same amino acid sequence and truncation of the polymerase gene (Figure S8).

For sample HBV10, further variant analysis was performed on the read mapping to investigate the possible presence of HBV quasispecies subpopulation. Table 4 describes the number of viral variants at different frequencies identified in the P and surface genes with at least 800× coverage for HBV 10. Filtering parameters were applied and variants identified in <20% of reads at a position were attributed to sequencing error. Variants detected in over 80% of sequenced reads were designated as static nucleotide changes contributing to the HBV genotype C for this sample. We observed a high proportion of variants at frequencies of >20–80% (17%, 37%, and 86%) for polymerase, large surface, and middle surface coding sequences, respectively. This finding indicates the potential presence of at least one quasispecies population for this sample. 

## 4. Discussion

In this study, we genotyped 14 HBV sequences from Australian blood donors. We found genotype C to be predominant, followed by genotypes B and D, then genotype A. This observation was consistent with previous findings that genotypes B and C are most prevalent in Australia, followed by genotypes A and D [17,47]. 

Whole genome sequences were obtained for four HBV isolates with the highest viral titres, and these were genotyped B or C. Discordance in genotypes predicted by analysis of large surface/polymerase genes and core genes can be indicative of recombination events. Three of the four whole genome sequences had recombination events between genotypes B and C in the core region. This finding explains why the HBV genotypes of these samples determined by HBV large surface/polymerase gene-based phylogenies were discordant with genotypes determined by phylogenetic analysis of HBV core gene, but in agreement with that of genotyping by HBV whole genomes. The discordance in genotypes observed in our study highlights potential inaccuracies if the genotype of a HBV isolate is determined by phylogenies of partial or short HBV sequences. Analysis of recombination and sequence diversity along HBV complete genomes or longer sequences can overcome the potential bias in genotyping HBV variations between different samples.

We were able to obtain partial sequences for 11 isolates in our sample set. The inability to obtain whole genomes for these samples is likely due to a combination of HBV genome structure, viral loads and primers used for DNA amplification. In the original study by Chook et al., successful amplification of HBV sequences was dependent on the amount of viral nucleic acid [31]. Low viral loads for many of our samples likely compromised amplification efficacy. We found that primer set 3 was the most successful at sequence amplification, as this primer set targets a short and structurally non-complex part of the genome. Primer set 4 targets the region where the minus strand has a short 5′ terminal redundancy attached with polymerase protein. Meanwhile, primer sets 1 and 2 are designed to target the region of HBV where the plus strand is not yet synthesised [31,48]. 

A previous study identified the polymerase and pre-surface/surface loci as the most genetically diverse regions along the HBV viral genome [47]. In this study we found two genetically diverse regions, nt103–203 and nt333–438, which appear to contain or overlap with sequences coding for two corresponding epitopes of HBsAg (AA51–70 and AA110–147 for wild-type HBsAg GQ183486) predicted by Rezaee et al. (2016) [26]. This observation was confirmed as sequences coding for the major antigenic segments of HBsAg from our samples were predicted to locate within or overlap with genetically diverse regions and as the segments were similar to those predicted by Rezaee et al. (2016) [26], We observed that the area of greatest diversity within the surface gene, in both Australian and non-Australian donor HBV isolates, appeared to incorporate the ‘a’ determinant of HBsAg. The ‘a’ determinant in the MHR is an important region of HBsAg, as mutations within this region have been reported to affect HBV surface antigenicity [49], cross-genotype vaccine protection [50] and efficacy of serological HBsAg detection assays [25,51]. We found that, generally, surface gene nucleotide diversity was lower in our Australian donor HBV sequences when compared to those from non-Australian donors. However, it is still unclear whether HBV surface gene nucleotide variations leading to different genotypes contributes to structural changes in HBsAg. Additional studies are required to understand the possible association between genotypic differences of HBV surface gene and the structure and function of HBsAg, and how this impacts the effectiveness of HBsAg serological screening between different genotypes.

We observed interesting nucleotide variations in the polymerase, large and middle surface genes for two HBV isolates from our samples. A nucleotide deletion in the region of polymerase and surface gene overlap resulted in frameshift and truncation of the polymerase and large/middle surface amino acid sequences in two samples. As the deletion occurred upstream of the small surface coding region, there was no impact on the expression of the small S protein, the target of HBsAg serology assays, and the two samples remained HBsAg-positive. Additionally, we identified a potential subpopulation of HBV quasispecies in one of these samples. Due to the presence of potential HBV quasispecies in this sample, we suspect that the donor may have had a long term, chronic HBV infection [52].

This study was limited by the low number of samples obtained from Australian blood donors, resulting in the inclusion of only eight HBV surface sequences of genotypes A–D from our samples. One possible reason for this is that Australia has a low estimated prevalence of chronic hepatitis B infection (0.9% in 2016) [53]. Moreover, blood donors are a low-risk population with regards to HBV infection, due to extensive pre-donation screening. Further studies on HBV genotypes and diversity with greater sample numbers should be conducted, although recruiting HBV-positive blood donors would be difficult considering the very low prevalence of HBV infections in Australia.

## 5. Conclusions

In summary, this study provides a snapshot of HBV genotypes circulating within the Australian blood donors that have not been previously investigated. It also provides a comparative insight highlighting a lower nucleotide diversity of the HBV surface gene from Australian donors compared to international blood donors. Overall, HBV genotypes A–D were found in Australian donor samples. Sequence analysis identified a region surrounding the ‘a’ determinant that displayed higher levels of nucleotide variability compared with other regions of the surface gene. Two samples were observed with truncated polymerase and large/middle surface genes, and one sample with potential HBV quasispecies was identified. 

## Figures and Tables

**Table 1:** Characteristics of the HBV plasma samples *.

Sample	Date Collected	HIV-1/HCV/HBV Multiplex NAT	HBV Discriminatory NAT	HCV Discriminatory NAT	HIV-1 Discriminatory NAT	Total Anti-HBc	Anti-HBc IgM	Anti-HBe	HbeAg	Total Anti-HBs	HBsAg
HBV 1	12-Dec-97	*REAC*	*REAC*	NONREAC	NONREAC	*POS*	NEG	POS	NEG	NEG	*POS*
HBV 2	12-Dec-97	*REAC*	*REAC*	NONREAC	NONREAC	*POS*	NA	NA	NA	NA	*POS*
HBV 3	06-Apr-98	*REAC*	*REAC*	NONREAC	NONREAC	NA	NA	NA	NA	NA	*POS*
HBV 4	31-May-02	*REAC*	*REAC*	NONREAC	NONREAC	NA	NA	NA	NA	NA	*POS*
HBV 5	25-Sep-02	*REAC*	*REAC*	*REAC*	NONREAC	NA	NA	NA	NA	NA	*POS*
HBV 6	11-Oct-02	*REAC*	*REAC*	NONREAC	NONREAC	NA	NA	NA	NA	NA	*POS*
HBV 7	29-Jan-03	*REAC*	*REAC*	NONREAC	NONREAC	NA	NA	NA	NA	NA	*POS*
HBV 8	07-Dec-03	*REAC*	*REAC*	NONREAC	NONREAC	NA	NA	NA	NA	NA	*POS*
HBV 9	09-Feb-04	*REAC*	*REAC*	NONREAC	NONREAC	*POS*	NEG	*POS*	NEG	NEG	*POS*
HBV 10	05-Mar-04	*REAC*	*REAC*	NONREAC	NONREAC	*POS*	NEG	*POS*	NEG	NEG	*POS*
HBV 11	11-Mar-04	*REAC*	*REAC*	NONREAC	NONREAC	*POS*	NEG	*POS*	NEG	NEG	*POS*
HBV 12	01-Apr-04	*REAC*	*REAC*	NONREAC	INVALID	NA	NA	NA	NA	NA	*POS*
HBV 13	20-May-04	*REAC*	*REAC*	NONREAC	NONREAC	NA	NA	NA	NA	NA	*POS*
HBV 14	05-Jul-04	*REAC*	*REAC*	NONREAC	NONREAC	NA	NA	NA	NA	NA	*POS*
HBV 15	14-Apr-11	*REAC*	*REAC*	NONREAC	NONREAC	*POS*	NA	NA	NA	NEG	*POS*
HBV 16	26-May-11	*REAC*	*REAC*	NONREAC	NONREAC	*POS*	NA	NA	NA	NEG	*POS*

* REAC—reactive, NONREAC—not reactive, POS—positive, NEG—negative, NA—not provided.

**Table 2:** HBV genotypes of study samples determined by phylogenetic analysis.

	**Genotype**	Determined by the Phylogeny of Core Genes	Determined by the Phylogeny of Polymerase/Large Surface Genes	Determined by Phylogeny of Whole Genomes	Consensus Genotype
Sample					
HBV 1		D	N/A *	N/A *	D +
HBV 2		C	N/A *	N/A *	C +
HBV 3		C	B	B	B
HBV 4		D	D	N/A *	D
HBV 5		N/A *	N/A *	N/A *	N/A *
HBV 6		C	N/A *	N/A *	C +
HBV 7		C	B	B	B
HBV 8		N/A *	N/A *	N/A *	N/A *
HBV 9		A	A	N/A *	A
HBV 10		C	C	N/A *	C
HBV 11		C	N/A *	N/A *	C +
HBV 12		C	C	C	C
HBV 13		C	B	B	B
HBV 14		D	N/A *	N/A *	D +
HBV 15		A	A	N/A *	A
HBV 16		C	N/A *	N/A *	C +

* N/A—the region was unsuccessfully amplified and sequenced; + genotypes were determined only by phylogenies of core genes.

**Table 3:** Number of sSNV and nsSNV identified inside the polymerase and large/middle/small surface coding sequences of HBV sequences from our samples, compared to the NCBI reference sequence.

Sample	Sequence Length (bp)/Genotype	Polymerase Sequences			Large Surface Sequences			Middle Surface Sequences			Small Surface Sequences		
		Total SNV/Length (bp)	sSNV	nsSNV	Total SNV/Length (bp)	sSNV	nsSNV	Total SNV/Length (bp)	sSNV	nsSNV	Total SNV/Length (bp)	sSNV	nsSNV
HBV 1	945/D	N/A			N/A			N/A			N/A		
HBV 2	1910/C	N/A			N/A			N/A			N/A		
HBV 3	3182/B *	198/2499	112	86	87/1170	49	38	52/846	24	28	32/681	13	20
HBV 4	3089/D	93/2499	60	33	30/1170	15	15	17/846	7	10	11/681	6	5
HBV 5	1530	N/A			N/A			N/A			N/A		
HBV 6	947/C	N/A			N/A			N/A			N/A		
HBV 7	3182/B *	201/2499	118	83	93/1170	52	41	57/846	26	31	40/681	17	23
HBV 8	N/A	N/A			N/A			N/A			N/A		
HBV 9	3100/A	198/2511	104	94	79/1170	39	40	48/846	19	29	31/681	13	18
HBV 10	3080/C	122/1710	57	65	36/420	26	10	11/96	5	6	25/681	10	15
HBV 11	947/C	N/A			N/A			N/A			N/A		
HBV 12	3181/C *	133/1710	59	11	67/1170	38	29	41/846	18	23	26/681	9	17
HBV 13	3197/B *	208/2514	116	92	98/1170	55	43	61/846	27	34	37/681	16	21
HBV 14	1910/D	N/A			N/A			N/A			N/A		
HBV 15	3093/A	196/2505	108	88	77/1170	44	10	44/846	19	25	29/681	13	16
HBV 16	939/C	N/A			N/A			N/A			N/A		

* Complete genome; N/A - polymerase or surface coding sequences were not available.

**Table 4:** sSNV and nsSNV frequencies from the polymerase and surface coding regions of sample HBV 10.

Variant Frequency	Polymerase Gene			Large Surface Gene			Middle Surface Gene			Small Surface Gene		
	Total	sSNV	nsSNV	Total	sSNV	nsSNV	Total	sSNV	nsSNV	Total	sSNV	nsSNV
>20–80%	20 (17%)	5	15	15 (37%)	6	9	12 (86%)	4	8	1 (5%)	0	1
>80–100%	97 (83%)	51	46	26 (63%)	22	4	2 (14%)	2	0	20 (95%)	9	11
Total	117	56	61	41	37	30	14	12	15	21	9	15

## Data Availability

Genbank accession numbers for the HBV sequences from our Australian blood donors are MW847929–MW847943.

## Supplementary Materials

Supplementary Materials can be found at https://www.mdpi.com/article/10.3390/v13071275/s1. Table S1: NCBI HBV reference sequences from blood donors used in this study, Table S2: Viral quantification of the HBV plasma samples, Figure S1: FastTree maximum likelihood tree built from HBV core genes from blood donors, Figure S2: FastTree maximum likelihood tree built from HBV polymerase genes from blood donors, Figure S3: FastTree maximum likelihood tree built from HBV large surface genes from blood donors, Figure S4: FastTree maximum likelihood tree built from HBV whole genomes from blood donors, Figure S5: Truncated polymerase amino acid sequence with frameshift mutation for HBV10 compared to the reference NC_003977, Figure S6: Truncated large surface amino acid sequence with frameshift mutation for HBV10 compared to the reference NC_003977, Figure S7: Truncated middle surface amino acid sequence with frameshift mutation for HBV10 compared to the reference NC_003977, Figure S8: Truncated polymerase amino acid sequence with frameshift mutation for HBV12 compared to the reference NC_003977 due to deletion of a nucleotide at either of two possible positions identified by mapping or by consensus sequence, File S1: Identification of known OBI related amino acid variants of HBsAg amongst blood donors, File S2: Bepipred linear epitope prediction for translated HBsAg from HBV 3, File S3: Bepipred linear epitope prediction for translated HBsAg from HBV 9, File S4: Bepipred linear epitope prediction for translated HBsAg from HBV 10.
