# Supplementary material for: Low Genetic Diversity of Hepatitis B Virus Surface Gene amongst Australian Blood Donors
Source: Viruses. 2021 Jun 30;13(7):1275. doi: 10.3390/v13071275 (PMC8310342; doi:10.3390/v13071275)
Supplement: Supplementary file 1 [file viruses-13-01275-s001.zip › File S3_Bepipred linear epitope prediction for translated HBsAg from HBV 9.pdf]

IEDB Analysis Resource

Home | Help | Example | Reference | Download | Contact

Bepipred Linear Epitope Prediction Results

Input Sequences

```
  1 MENITSGFLG PLLVLQAGFF SLTKILTIPQ SLDSWWTSLS FLGGAPVCLG QNSQSPTSNH
 61 SPTSCPPICP GYRWMCLRRF IIFLFILLLC LIFLLVLLDY QGMLPVCPLI PGSTTTSTGP
121 CKTCTTPAQG NSMFPSCCCT KPTDGNCTCI PIPSSWAFAK YLWEWASVRF SWLSLLVPFV
181 QWFVGLSPTV WLSAIWMMWY WGPSLYSIVR PFIPLLPIFF CLWVYI
```

Center position: 4 **Threshold:** 0.350 Recalculate

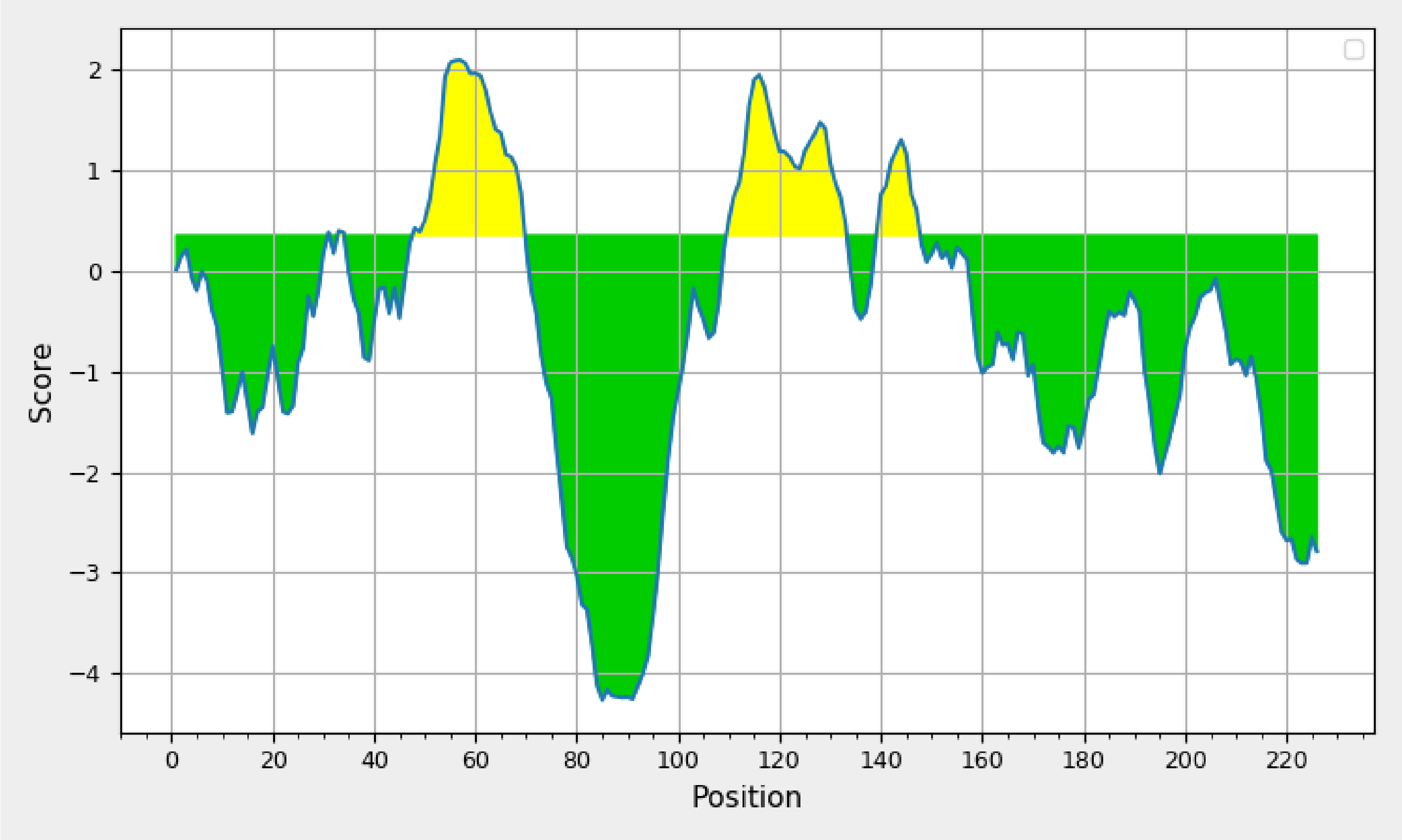

Average: -0.581 **Minimum:** -0.008 **Maximum:** 2.092

Predicted peptides:

No.	Start	End	Peptide	Length	
1	31	31	S	1	
2	33	34	DS	2	
3	48	69	CLGQNSQSPTSNHSPTSCPPIC	22	~nt142-207
4	110	133	IPGSTTTSTGPCKTCTTPAQGNSM	24	~nt328-399
5	140	147	TKPTDGNC	8	~nt418-441

Predicted residue scores:

Position	Residue	Score	Assignment
1	M	0.012	.
2	E	0.147	.
3	N	0.208	.

Position	Residue	Score	Assignment
4	I	-0.068	.
5	T	-0.198	.
6	S	-0.008	.
7	G	-0.093	.
8	F	-0.391	.
9	L	-0.555	.
10	G	-0.975	.
11	P	-1.408	.
12	L	-1.399	.
13	L	-1.213	.
14	V	-1.007	.
15	L	-1.316	.
16	Q	-1.615	.
17	A	-1.401	.
18	G	-1.356	.
19	F	-1.041	.
20	F	-0.749	.
21	S	-1.078	.
22	L	-1.397	.
23	T	-1.419	.
24	K	-1.343	.
25	I	-0.911	.
26	L	-0.767	.
27	T	-0.250	.
28	I	-0.453	.
29	P	-0.200	.
30	Q	0.168	.
31	S	0.381	E
32	L	0.174	.
33	D	0.398	E
34	S	0.380	E
35	W	-0.027	.
36	W	-0.286	.
37	T	-0.426	.
38	S	-0.862	.
39	L	-0.890	.
40	S	-0.507	.
41	F	-0.180	.
42	L	-0.167	.
43	G	-0.425	.
44	G	-0.169	.
45	A	-0.473	.
46	P	-0.109	.
47	V	0.268	.
48	C	0.425	E
49	L	0.386	E
50	G	0.499	E

Position	Residue	Score	Assignment
51	Q	0.709	E
52	N	1.058	E
53	S	1.338	E
54	Q	1.926	E
55	S	2.066	E
56	P	2.087	E
57	T	2.092	E
58	S	2.058	E
59	N	1.958	E
60	H	1.963	E
61	S	1.934	E
62	P	1.794	E
63	T	1.570	E
64	S	1.401	E
65	C	1.367	E
66	P	1.154	E
67	P	1.133	E
68	I	1.029	E
69	C	0.767	E
70	P	0.228	.
71	G	-0.198	.
72	Y	-0.426	.
73	R	-0.855	.
74	W	-1.120	.
75	M	-1.270	.
76	C	-1.799	.
77	L	-2.270	.
78	R	-2.744	.
79	R	-2.847	.
80	F	-3.026	.
81	I	-3.317	.
82	I	-3.363	.
83	F	-3.721	.
84	L	-4.121	.
85	F	-4.264	.
86	I	-4.162	.
87	L	-4.222	.
88	L	-4.230	.
89	L	-4.238	.
90	C	-4.234	.
91	L	-4.251	.
92	I	-4.129	.
93	F	-4.006	.
94	L	-3.830	.
95	L	-3.437	.
96	V	-2.985	.
97	L	-2.388	.

Position	Residue	Score	Assignment
98	L	-1.872	.
99	D	-1.451	.
100	Y	-1.190	.
101	Q	-0.914	.
102	G	-0.575	.
103	M	-0.175	.
104	L	-0.360	.
105	P	-0.497	.
106	V	-0.667	.
107	C	-0.616	.
108	P	-0.329	.
109	L	0.212	.
110	I	0.512	E
111	P	0.744	E
112	G	0.870	E
113	S	1.173	E
114	T	1.645	E
115	T	1.899	E
116	T	1.944	E
117	S	1.824	E
118	T	1.591	E
119	G	1.371	E
120	P	1.188	E
121	C	1.181	E
122	K	1.128	E
123	T	1.034	E
124	C	1.016	E
125	T	1.191	E
126	T	1.283	E
127	P	1.367	E
128	A	1.474	E
129	Q	1.409	E
130	G	1.064	E
131	N	0.874	E
132	S	0.734	E
133	M	0.470	E
134	F	0.003	.
135	P	-0.384	.
136	S	-0.480	.
137	C	-0.406	.
138	C	-0.134	.
139	C	0.329	.
140	T	0.758	E
141	K	0.843	E
142	P	1.084	E
143	T	1.194	E
144	D	1.303	E

Position	Residue	Score	Assignment
145	G	1.160	E
146	N	0.753	E
147	C	0.609	E
148	T	0.262	.
149	C	0.087	.
150	I	0.168	.
151	P	0.281	.
152	I	0.126	.
153	P	0.188	.
154	S	0.026	.
155	S	0.234	.
156	W	0.167	.
157	A	0.111	.
158	F	-0.381	.
159	A	-0.856	.
160	K	-1.014	.
161	Y	-0.959	.
162	L	-0.931	.
163	W	-0.609	.
164	E	-0.736	.
165	W	-0.720	.
166	A	-0.885	.
167	S	-0.606	.
168	V	-0.626	.
169	R	-1.043	.
170	F	-0.931	.
171	S	-1.361	.
172	W	-1.709	.
173	L	-1.749	.
174	S	-1.811	.
175	L	-1.743	.
176	L	-1.809	.
177	V	-1.539	.
178	P	-1.560	.
179	F	-1.761	.
180	V	-1.554	.
181	Q	-1.283	.
182	W	-1.225	.
183	F	-0.933	.
184	V	-0.653	.
185	G	-0.405	.
186	L	-0.458	.
187	S	-0.412	.
188	P	-0.444	.
189	T	-0.207	.
190	V	-0.296	.
191	W	-0.407	.

Position	Residue	Score	Assignment
192	L	-1.001	.
193	S	-1.325	.
194	A	-1.745	.
195	I	-2.012	.
196	W	-1.833	.
197	M	-1.647	.
198	M	-1.441	.
199	W	-1.237	.
200	Y	-0.772	.
201	W	-0.558	.
202	G	-0.451	.
203	P	-0.265	.
204	S	-0.218	.
205	L	-0.195	.
206	Y	-0.080	.
207	S	-0.340	.
208	I	-0.584	.
209	V	-0.931	.
210	R	-0.877	.
211	P	-0.903	.
212	F	-1.043	.
213	I	-0.851	.
214	P	-1.068	.
215	L	-1.411	.
216	L	-1.884	.
217	P	-1.988	.
218	I	-2.281	.
219	F	-2.590	.
220	F	-2.676	.
221	C	-2.665	.
222	L	-2.868	.
223	W	-2.903	.
224	V	-2.902	.
225	Y	-2.647	.
226	I	-2.787	.

Download result

© 2005-2021 | IEDB Home
Supported by a contract from the National Institute of Allergy and Infectious Diseases, a component of the National Institutes of Health in the Department of Health and Human Services.
