# Supplementary material for: Low Genetic Diversity of Hepatitis B Virus Surface Gene amongst Australian Blood Donors
Source: Viruses. 2021 Jun 30;13(7):1275. doi: 10.3390/v13071275 (PMC8310342; doi:10.3390/v13071275)
Supplement: Supplementary file 1 [file viruses-13-01275-s001.zip › File S4_Bepipred linear epitope prediction for translated HBsAg from HBV 10.pdf]

IEDB Analysis Resource

Home | Help | Example | Reference | Download | Contact

Bepipred Linear Epitope Prediction Results

Input Sequences

```
  1 MESTTSGFLG PLLVLQAGFF LLTRILTIPQ SLDSWWTSLN FLGGAPTCPG QNLQSPTSNH
 61 SPTSCPPICP GYRWMCLRRF IIFLFILLLC LIFLLVLLDY QGMLPVCPLL PGTSTTSTGP
121 CKTCTTPAQG TSMFPSCCCT KPSDGNCTCI PIPSSWAFAR FLWEWASVRF SWLSLLVPFV
181 QWFAGLSPTV WLSVIWMMWY WGPSLYNILK PFLPLLPIFF CLWVYI
```

Center position: 4 **Threshold:** 0.350 Recalculate

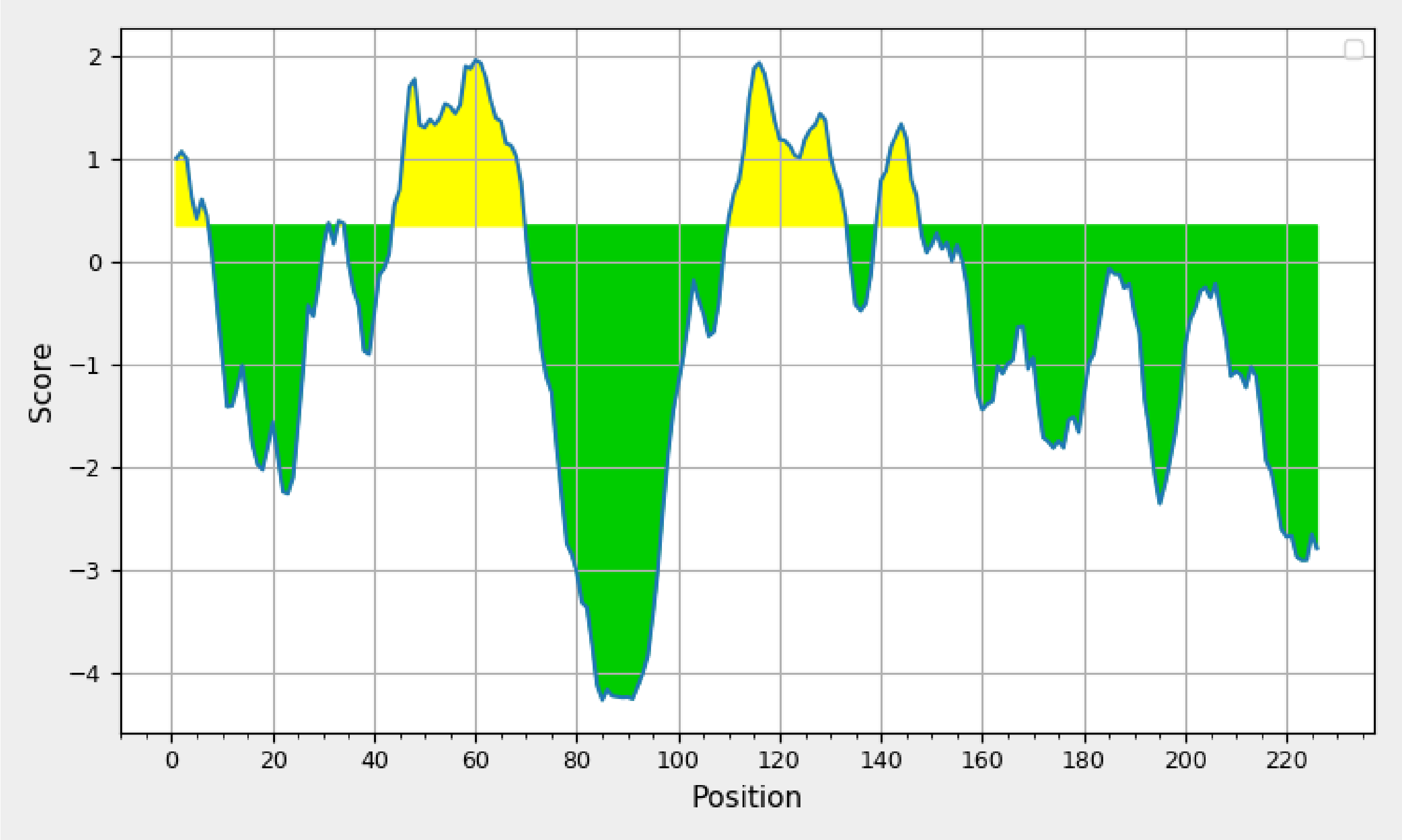

Average: -0.585 **Minimum:** -0.031 **Maximum:** 1.963

Predicted peptides:

No.	Start	End	Peptide	Length	
1	1	7	MESTTSG	7	
2	31	31	S	1	
3	33	34	DS	2	
4	44	69	GAPTCPGQNLQSPTSNHSPTSCPPIC	26	~nt130-207
5	110	133	LPGTSTTSTGPCKTCTTPAQGTSM	24	~nt328-399
6	139	147	CTKPSDGNC	9	~nt415-441

Predicted residue scores:

Position	Residue	Score	Assignment
1	M	1.007	E
2	E	1.073	E

Position	Residue	Score	Assignment
3	S	1.002	E
4	T	0.627	E
5	T	0.420	E
6	S	0.606	E
7	G	0.449	E
8	F	0.090	.
9	L	-0.418	.
10	G	-0.910	.
11	P	-1.408	.
12	L	-1.399	.
13	L	-1.213	.
14	V	-1.007	.
15	L	-1.396	.
16	Q	-1.774	.
17	A	-1.969	.
18	G	-2.023	.
19	F	-1.802	.
20	F	-1.556	.
21	L	-1.901	.
22	L	-2.235	.
23	T	-2.257	.
24	R	-2.102	.
25	I	-1.590	.
26	L	-1.037	.
27	T	-0.422	.
28	I	-0.530	.
29	P	-0.232	.
30	Q	0.153	.
31	S	0.381	E
32	L	0.174	.
33	D	0.398	E
34	S	0.376	E
35	W	-0.034	.
36	W	-0.284	.
37	T	-0.429	.
38	S	-0.868	.
39	L	-0.897	.
40	N	-0.514	.
41	F	-0.130	.
42	L	-0.063	.
43	G	0.083	.
44	G	0.554	E
45	A	0.696	E
46	P	1.231	E
47	T	1.701	E
48	C	1.778	E
49	P	1.331	E

Position	Residue	Score	Assignment
50	G	1.307	E
51	Q	1.386	E
52	N	1.335	E
53	L	1.402	E
54	Q	1.537	E
55	S	1.511	E
56	P	1.442	E
57	T	1.527	E
58	S	1.901	E
59	N	1.881	E
60	H	1.963	E
61	S	1.934	E
62	P	1.794	E
63	T	1.570	E
64	S	1.401	E
65	C	1.367	E
66	P	1.154	E
67	P	1.133	E
68	I	1.029	E
69	C	0.767	E
70	P	0.228	.
71	G	-0.198	.
72	Y	-0.426	.
73	R	-0.855	.
74	W	-1.120	.
75	M	-1.270	.
76	C	-1.799	.
77	L	-2.270	.
78	R	-2.744	.
79	R	-2.847	.
80	F	-3.026	.
81	I	-3.317	.
82	I	-3.363	.
83	F	-3.721	.
84	L	-4.121	.
85	F	-4.264	.
86	I	-4.162	.
87	L	-4.222	.
88	L	-4.230	.
89	L	-4.238	.
90	C	-4.234	.
91	L	-4.251	.
92	I	-4.129	.
93	F	-4.006	.
94	L	-3.830	.
95	L	-3.437	.
96	V	-2.985	.

Position	Residue	Score	Assignment
97	L	-2.388	.
98	L	-1.872	.
99	D	-1.451	.
100	Y	-1.190	.
101	Q	-0.914	.
102	G	-0.575	.
103	M	-0.175	.
104	L	-0.368	.
105	P	-0.513	.
106	V	-0.723	.
107	C	-0.684	.
108	P	-0.408	.
109	L	0.110	.
110	L	0.436	E
111	P	0.666	E
112	G	0.795	E
113	T	1.107	E
114	S	1.587	E
115	T	1.882	E
116	T	1.938	E
117	S	1.828	E
118	T	1.619	E
119	G	1.373	E
120	P	1.191	E
121	C	1.181	E
122	K	1.128	E
123	T	1.034	E
124	C	1.016	E
125	T	1.193	E
126	T	1.284	E
127	P	1.332	E
128	A	1.442	E
129	Q	1.376	E
130	G	1.032	E
131	T	0.842	E
132	S	0.702	E
133	M	0.438	E
134	F	-0.031	.
135	P	-0.417	.
136	S	-0.477	.
137	C	-0.404	.
138	C	-0.128	.
139	C	0.362	E
140	T	0.794	E
141	K	0.882	E
142	P	1.122	E
143	S	1.233	E

Position	Residue	Score	Assignment
144	D	1.341	E
145	G	1.199	E
146	N	0.790	E
147	C	0.642	E
148	T	0.267	.
149	C	0.090	.
150	I	0.168	.
151	P	0.281	.
152	I	0.126	.
153	P	0.188	.
154	S	0.007	.
155	S	0.163	.
156	W	0.015	.
157	A	-0.239	.
158	F	-0.776	.
159	A	-1.280	.
160	R	-1.438	.
161	F	-1.383	.
162	L	-1.355	.
163	W	-1.013	.
164	E	-1.089	.
165	W	-0.991	.
166	A	-0.957	.
167	S	-0.635	.
168	V	-0.626	.
169	R	-1.043	.
170	F	-0.931	.
171	S	-1.361	.
172	W	-1.709	.
173	L	-1.749	.
174	S	-1.811	.
175	L	-1.743	.
176	L	-1.809	.
177	V	-1.539	.
178	P	-1.511	.
179	F	-1.661	.
180	V	-1.307	.
181	Q	-0.989	.
182	W	-0.885	.
183	F	-0.593	.
184	A	-0.313	.
185	G	-0.065	.
186	L	-0.117	.
187	S	-0.121	.
188	P	-0.254	.
189	T	-0.214	.
190	V	-0.497	.

Position	Residue	Score	Assignment
191	W	-0.701	.
192	L	-1.341	.
193	S	-1.665	.
194	V	-2.085	.
195	I	-2.352	.
196	W	-2.173	.
197	M	-1.938	.
198	M	-1.681	.
199	W	-1.331	.
200	Y	-0.818	.
201	W	-0.562	.
202	G	-0.458	.
203	P	-0.286	.
204	S	-0.245	.
205	L	-0.350	.
206	Y	-0.214	.
207	N	-0.489	.
208	I	-0.726	.
209	L	-1.113	.
210	K	-1.065	.
211	P	-1.097	.
212	F	-1.223	.
213	L	-1.025	.
214	P	-1.113	.
215	L	-1.478	.
216	L	-1.934	.
217	P	-2.046	.
218	I	-2.299	.
219	F	-2.599	.
220	F	-2.676	.
221	C	-2.665	.
222	L	-2.868	.
223	W	-2.903	.
224	V	-2.902	.
225	Y	-2.647	.
226	I	-2.787	.

Download result

© 2005-2021 | IEDB Home
Supported by a contract from the National Institute of Allergy and Infectious Diseases, a component of the National Institutes of Health in the Department of Health and Human Services.
