# Supplementary material for: Low Genetic Diversity of Hepatitis B Virus Surface Gene amongst Australian Blood Donors
Source: Viruses. 2021 Jun 30;13(7):1275. doi: 10.3390/v13071275 (PMC8310342; doi:10.3390/v13071275)
Supplement: Supplementary file 1 [file viruses-13-01275-s001.zip › Supplementary materials for MDPI viruses.pdf]

Low genetic diversity of hepatitis B virus surface gene amongst Australian blood donors

Table S1. NCBI HBV reference sequences from blood donors used in this study

Genotype	References for genotyping by phylogenetic analysis	References for comparative analysis of HBV surface gene
A	AF090842	JN182329.1
	X02763	FJ904411.1
	X51970	GQ477504.1
		GQ477466.1
		GQ477484.1
		GQ477498.1
		GQ477474.1
		LC458430.1
		LC458431.1
		GQ161813.1
		GQ477497.1
		GQ477471.1
		GQ477463.1
		GQ477469.1
		GQ477464.1
		FJ904434.1
		GQ477491.1
		GQ477486.1
		GQ477482.1
		GQ477478.1
		GQ477475.1
		GQ477472.1
		GQ477473.1
		JX310731.1
		JX310732.1
		JX310730.1
		JX310725.1
		JX310724.1
		FJ904390.2
		FJ904349.2
B	D00329	GQ924660.1
	AB073846	GQ924651.1
	AB602818	GQ924648.1
		GQ924645.1
		GQ924639.1
		KM359440.1
		GQ924656.1
		GQ924659.1
		GQ924654.1
		GQ924653.1
		GQ924646.1

		GQ924644.1
		GQ924641.1
		GQ924640.1
		GQ924638.1
		GQ924637.1
		GQ924634.1
		GQ924632.1
		GQ924630.1
		GQ924626.1
		GQ924625.1
		GQ924621.1
		GQ924617.1
		GQ924607.1
		GQ924606.1
		GQ924605.1
		GQ924624.1
		GQ924611.1
		KX276970.1
		KX276962.1
C	AB014381	GQ924657.1
	M12906	GQ924655.1
	X04615	GQ924642.1
		GQ924623.1
		GQ924604.1
		KM359441.1
		LC458432.1
		EF688062.1
		GQ924658.1
		GQ924650.1
		GQ924649.1
		GQ924643.1
		GQ924636.1
		GQ924633.1
		FJ904423.1
		GQ924622.1
		GQ924620.1
		GQ924619.1
		GQ924618.1
		GQ924616.1
		GQ924609.1
		GQ924613.1
		GQ924612.1
		KX276965.1
		KX276959.1
		KX276958.1
		KX276994.1
		KX276992.1
		KX276990.1
		KX276988.1
D	M32138	JF754635.1
	X65259	JF754633.1
		JF754623.1
		JF754614.1

	X85254	JF754600.1
		GU456684.1
		GU456682.1
		GU456666.1
		GU456655.1
		GU456646.1
		FJ904447.1
		FJ904442.1
		FJ904437.1
		FJ904430.1
		FJ904415.1
		FJ904394.1
		JN642165.1
		JN642163.1
		JN642159.1
		JN642156.1
		JN642153.1
		KM359442.1
		EU155895.1
		GQ477459.1
		GQ477457.1
		GQ477454.1
		GQ477456.1
		GQ477458.1
		EU155893.1
		AB033559.1
E	X75657	GQ161836.1
	AB032431	GQ161834.1
		GQ161824.1
		GQ161802.1
		GQ161793.1
		GQ161762.1
		GQ161755.1
		GQ161818.1
		GQ161821.1
		GQ161799.1
		GQ161805.1
		GQ161830.1
		GQ161827.1
		GQ161828.1
		GQ161825.1
		GQ161826.1
		GQ161819.1
		GQ161817.1
		GQ161816.1
		GQ161811.1
		HQ385261.1
		HQ385255.1
		HQ385252.1
		HQ385250.1
		HQ385248.1
		HQ385241.1
		HQ385239.1

		HQ385267.1<br>HQ385262.1<br>HQ385256.1
F	AB036910<br>AF223965<br>X69798	HM467760.1<br>HM467761.1<br>HM467762.1<br>HM467771.1<br>HM467772.1<br>HM467783.1<br>HM467784.1<br>HM467785.1<br>HM467786.1
G	AF405706<br>AB064310<br>AF160501	GU565217.1<br>HM467773.1<br>HM467770.1<br>HM467769.1
H	AY090454<br>AY090457<br>AY090460	KX372218.1
I	FJ023664<br>AB562463	Unavailable
J	AB486012	Unavailable

Table S2. Viral quantification of the HBV plasma samples

Sample	Viral load (gene copies/µL)
HBV 1	19
HBV 2	64
HBV 3	788,124
HBV 4	70
HBV 5	12
HBV 6	15
HBV 7	3,668,659
HBV 8	9
HBV 9	53
HBV 10	141
HBV 11	19
HBV 12	716,133
HBV 13	2,464
HBV 14	101
HBV 15	910
HBV 16	215 IU/mL (1,130 copies/ µL) *

(*) The result for HBV 16 was provided by Pathology Queensland of Queensland Health Clinical Statewide Services.

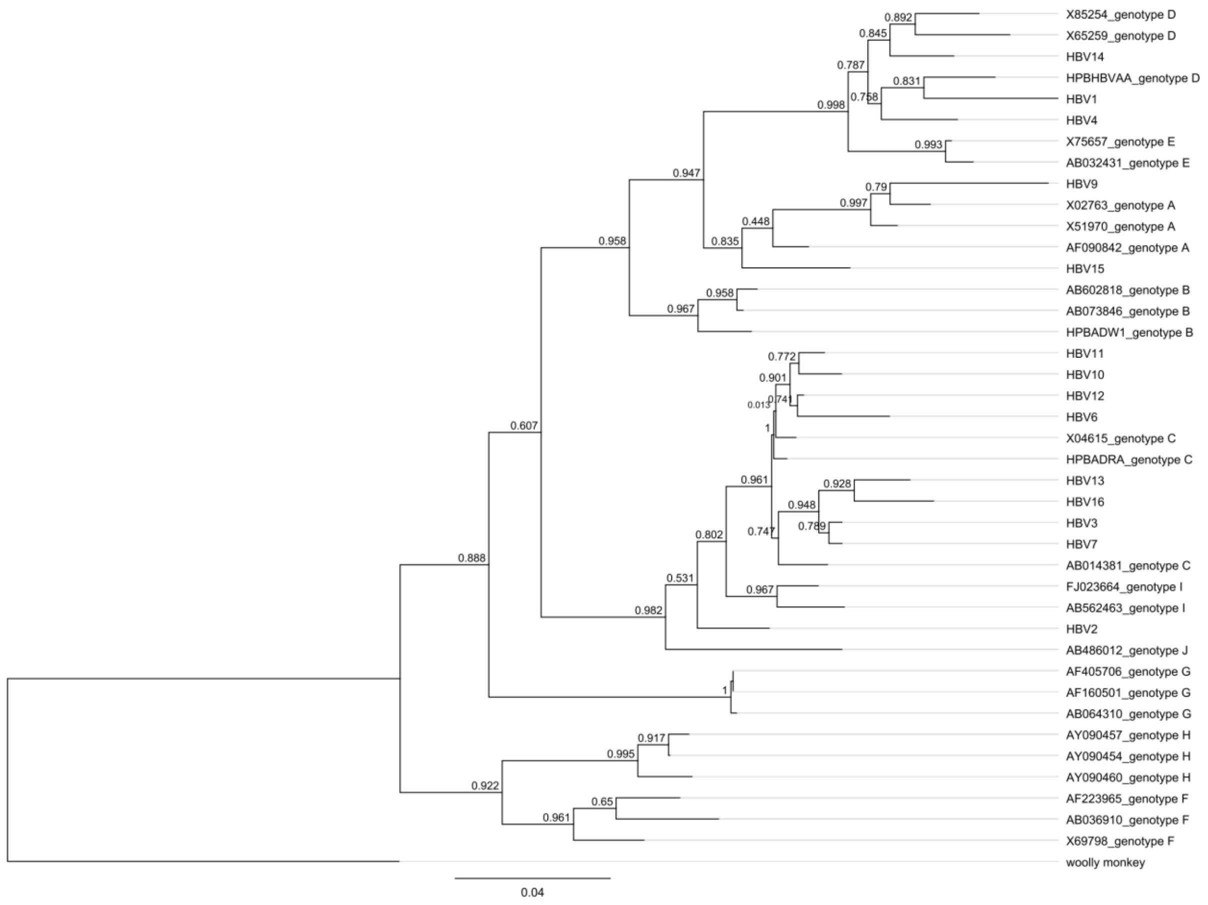

Figure S1. FastTree maximum likelihood tree built from HBV core genes from blood donors

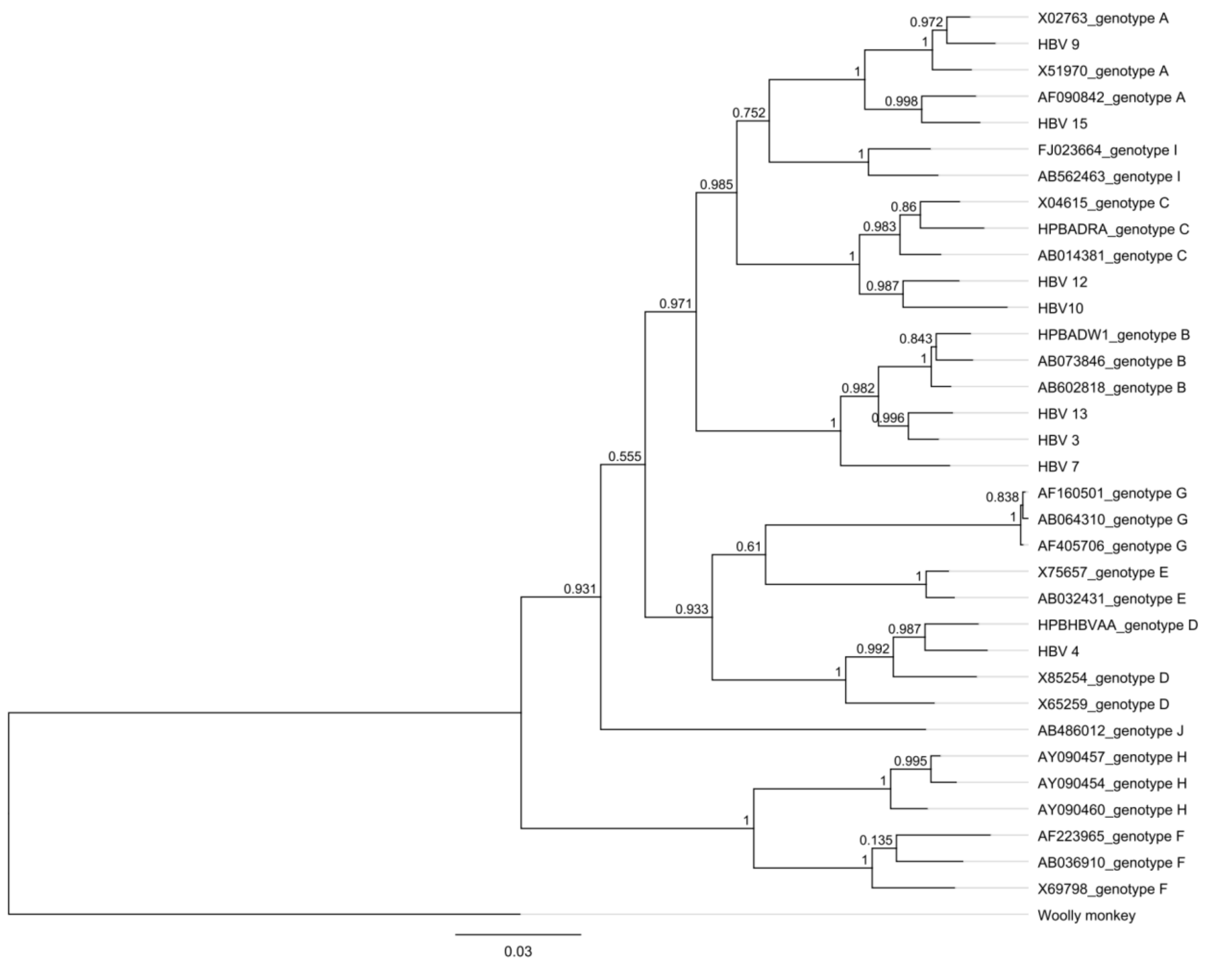

Figure S2. FastTree maximum likelihood tree built from HBV polymerase genes from blood donors

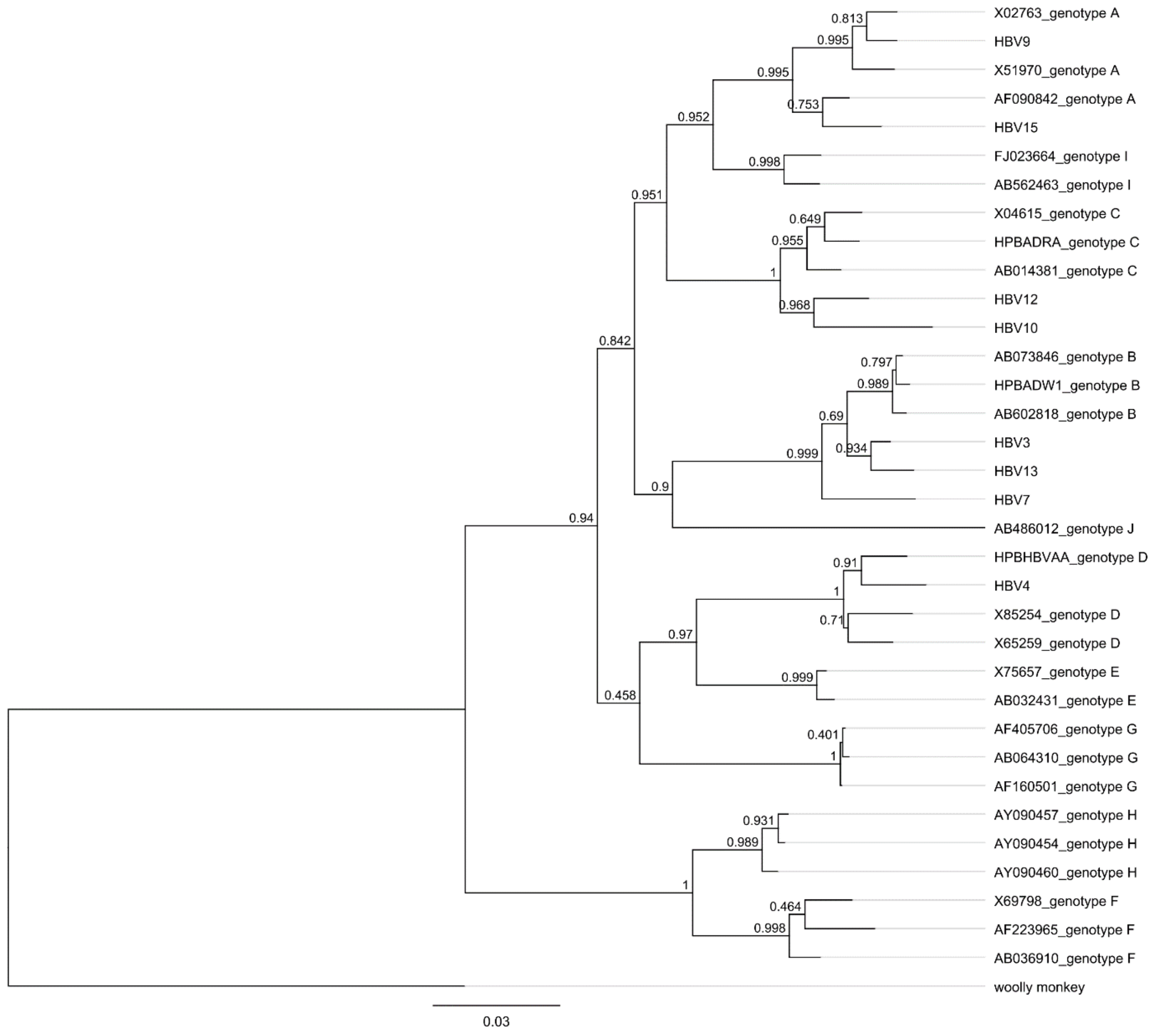

Figure S3. FastTree maximum likelihood tree built from HBV large surface genes from blood donors

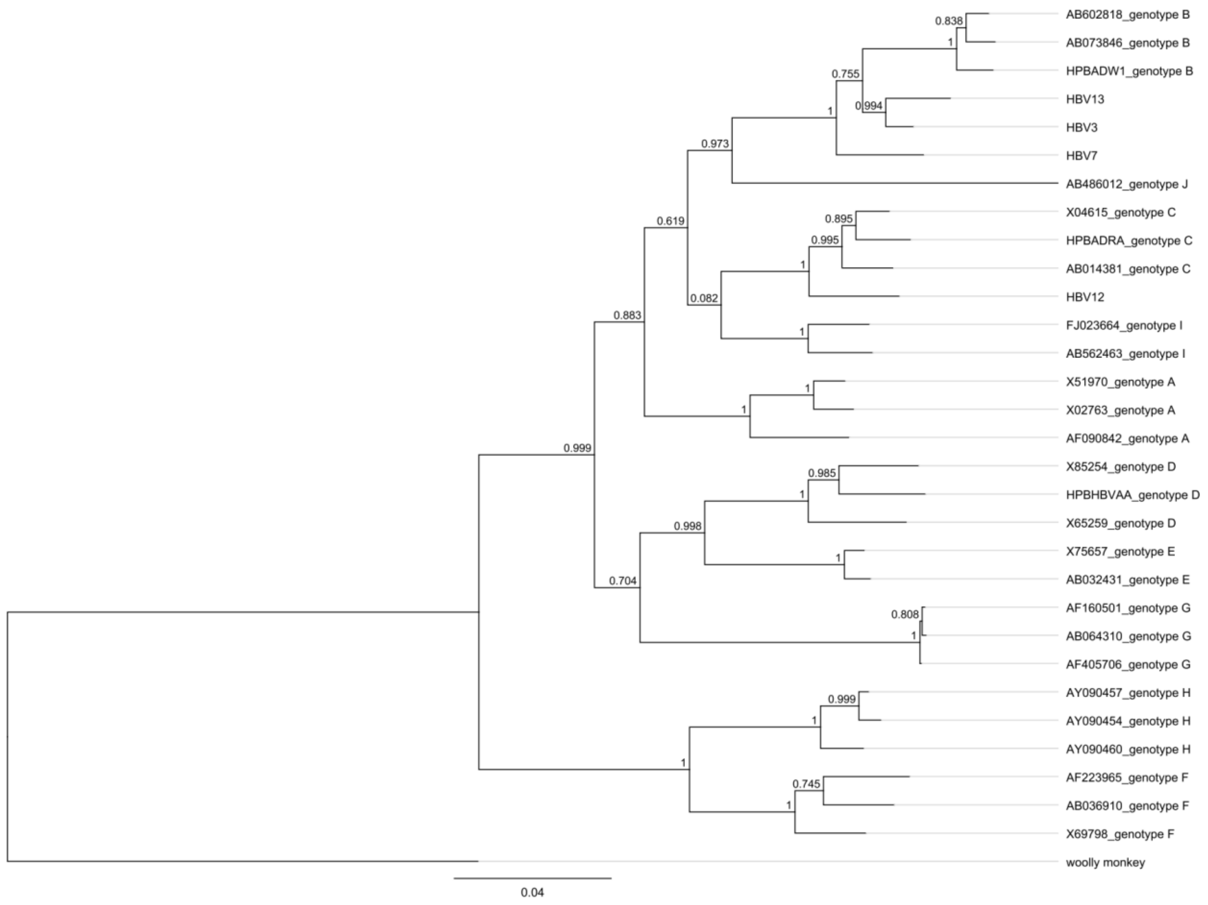

Figure S4. FastTree maximum likelihood tree built from HBV whole genomes from blood donors

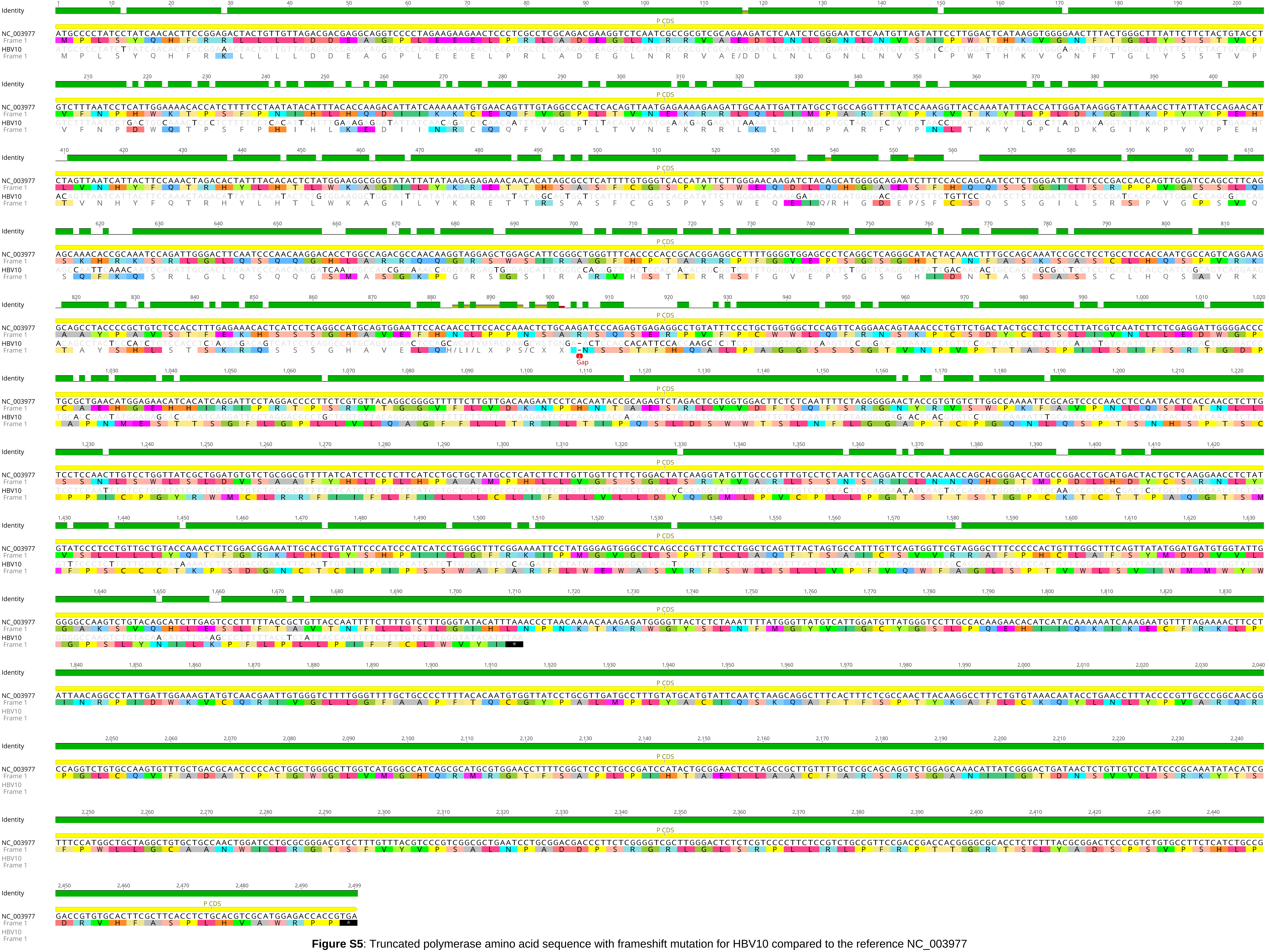

Figure S5: Truncated polymerase amino acid sequence with frameshift mutation for HBV10 compared to the reference NC_003977

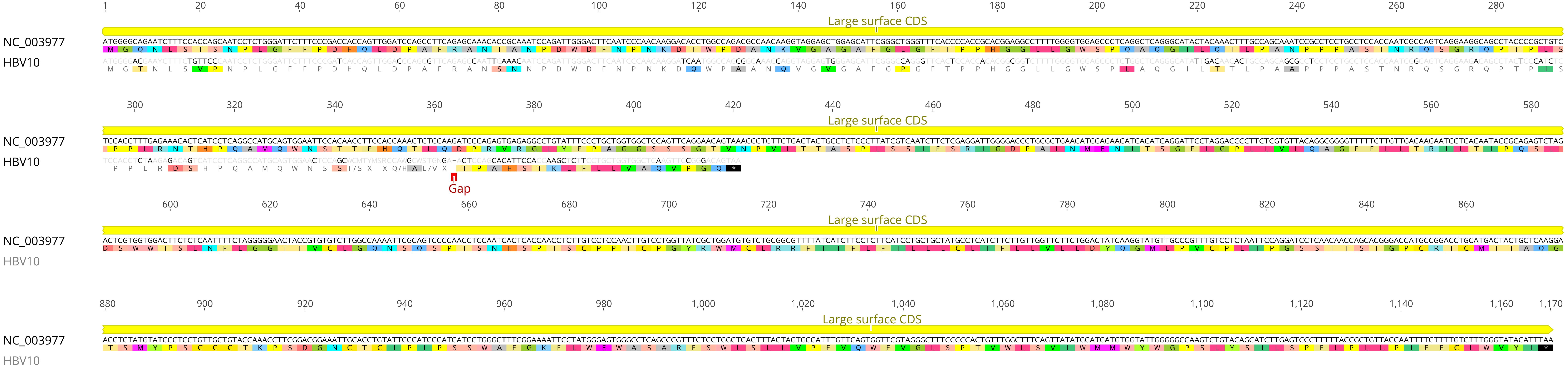

Figure S6: Truncated large surface amino acid sequence with frameshift mutation for HBV10 compared to the reference NC_003977

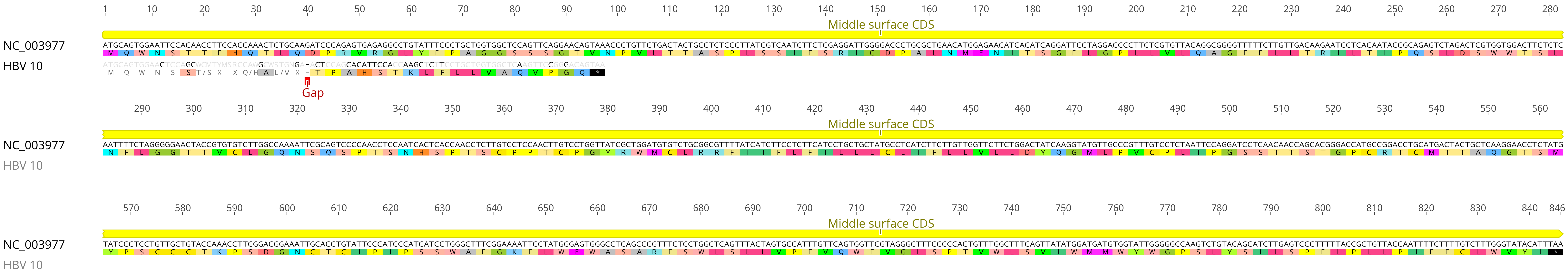

Figure S7: Truncated middle surface amino acid sequence with frameshift mutation for HBV10 compared to the reference NC_003977

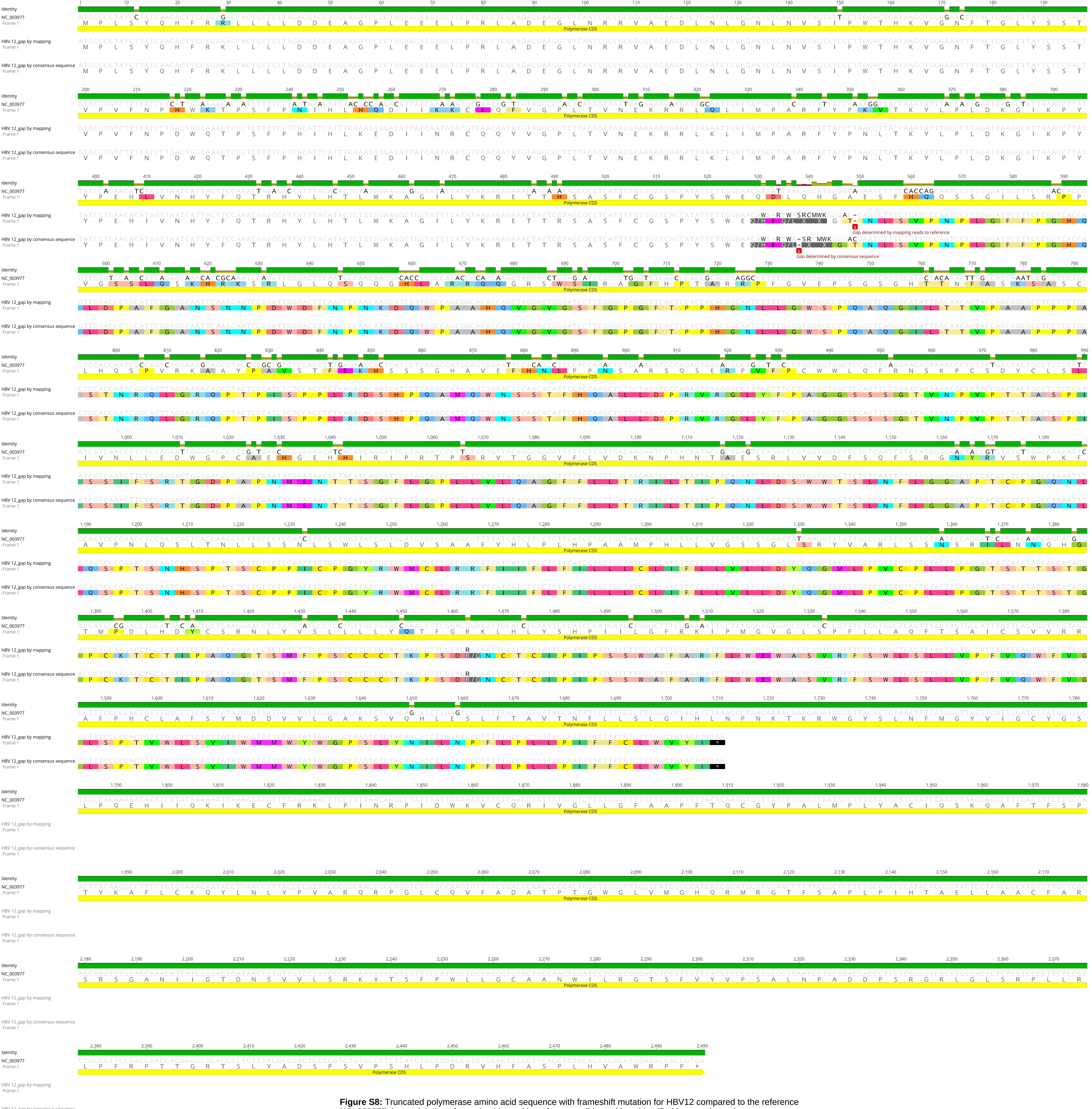

Figure S8: Truncated polymerase amino acid sequence with frameshift mutation for HBV12 compared to the reference NC_003977 due to deletion of a nucleotide at either of two possible positions identified by mapping or by consensus sequence
